# Labor

**Published:** 1861-10

**Authors:** W. H. Atkinson


DENTAL REGISTER OF THE WEST.

Vol. XV.] OCTOBER, 1861. [No. 10.

### Original Essays and Communications.

### LABOR.

BY W. H. ATKINSON, D. D. S.

[Read before the Indiana State Dental Association, Jan., 1861.]

Labor, to be eminently successful, must be regular and persistent. It matters not in what department of the activities of life the lot is cast, these principles are, like truths, immutable.

The divisions of labor naturally present themselves in a certain definite order.

I. Into receptive and projective.

II. ,0. Fractional--1. Unitary—2. Binary—3. Ternary—and 4. Multitudinous; each of which must be the result of receptive and projective forms of the various sorts and degrees.

*a.* Fractional, belonging to amorphous chaotic conditions. *b.* Unitary, to mineral. *c.* Binary, to vegetable. *d.* Ternary, to animal, and *e.* Multitudinous, to anthropologica. planes of labor.

The attributes of each plane will satisfactorily elucidate this to the attentive.

To fractional labors we must not look for entire attributive display; but particles and granules, without constant regular attribute of even fractional form, hold the dominion here.

To the mineral we may clearly perceive that definiteness of form—its sole (unitary) attribute—properly belongs, and is the basis of all corporeal, material shapes.

Vegetable—Binary-attribute, possessing not only definiteness of form, but has also the power of multiplication (the outcrop of sex displays itself between budding) and fructification, the first being only multiplying the same structure—the second being example of true reproduction.

The animal is ternary in attribute—possessing form, reproductive power, and added to these the essential one of intelligence, which enables it to be *conscious* of its own existence.

Anthropolgical plane of existences has multitudinous attribute, viz: Form, reproduction and consciousness—To which are added mental and moral attributes, themselves compounds, enclosing the possibles to the full measure of all these; to but name which would expose to the liability of being dubbed ideal, visionary, heterodox, etc., etc. And for the present it is probably wise to forbear to further pursue the "inevitable" line of march!

The great majority of labor, in this country, is performed for a consideration, and that of a money value, rather than as an expression on the part of the laborer that he is willing to spend and be spent for the sake of the high consciousness of being of *use* in the world, which really more ennobles a man than all other possessions beside. I need only to refer to the thousand little things performed in the every-day "courtesies" of life, that so potently bind society together in a sweet and lasting interchange of these, without fear or hope of reward, other than this very remuneration of the consciousness of having saved a fellow-creature from pain or loss of life or limb, or a soul from death.

Labor, cheerfully performed, elevates it to "exercise,"—from pain to pleasure, from unwilling drudgery to blissful recreation, which negatives the original sense of the Latin root, meaning to fail, give out, be exhausted, run down, like a clock, so as to require replenishing.

A well selected time and occasion have much to do with the efficiency of the various labors. As the day of twenty-four hours is our best unitary division of time, because including the entire round of phases, it will best serve us in our divisions to have especial reference to the natural provision of light and darkness, with their intermediate blendings, these two culminating points of this day corresponding to 12 o'clock at noon and 12 at night. They are also inceptive points, as well as points of focalization of the mutations of the two opposites—Light, and its absence we denominate Darkness.

To the *morning* especially belong projective labors; for the very good reason that when we are fully charged with the elements of capability, we the more easily accomplish exhaustive and beneficent works. Projective works are best done in strong light, and they should be entirely spontaneous.

The afternoon should be given up to receptive forms of labor, such as studies, visits of mercy, observations, etc., etc. In a word, learning—prehensively filling our mentals with the pabulum upon which to exercise our ruminating powers, so that we may become again charged for the next morning's repetition of projective labor. To properly execute these, they should be done in a receding light and with much meekness of spirit.

Commingled labors properly belong to the absence of light, and are embryonic—rumination, reflection, gestation, and recapitulation. If all these be properly turned over in regular succession, from one end of the chain to the other, and then back, so as to cause the whole number of its links to come again potently before the consciousness, we in some degree digest them, and lay them away for future use. But if we have been literally stuffed so full that the mentals were so clogged that we had not this room to recapitulate, on the law of memory, we are just in the condition of the gourmand who has so distended his stomach as to close by stretching the mucous and gastric follicles so tightly that the blood could not inflow to their delicate net-work, and produce the proper solvent of the contained mass, out of which to prepare the pabulum of life: and thus, instead of being recreated, is made sick, or poisoned upon the very thing that would have sustained and improved him, if only taken in moderation and with propriety. And who pities under such circumstances? *Ans.*—A very few who know how sweet to the taste it is to receive a fresh supply of new mental food; who alone are able to help out of the dilemma, by just quietly asking a few questions touching some of the incidents of labor; that like uncorking a bottle of champagne, gives it vent, and so relief is at once attained; by disposing of a link at a time, incident after incident, taking just what is adapted to the present use out of it, or passing it altogether, as effete matter, not worth the room it would occupy. And after thus disposing of the entire gorged mass of receptive labors, we may commit ourselves to our guardians, for the repose our worn machinery needs. As soon as the kind little angels of the life forces have filled our receptacles, we from this cause return to consciousness, and we say we are awake. Then if we would fitly do our work, let us not turn over and court sleep again; for if the proper steps, just rehearsed, have been taken, we will be more wasted than invigorated by a too full, crowded, stuffed state of projective power, that requires the master to teach us how to put again in the proper state of activity, to avoid spoiling the delicate brain centers, where ideas become thoughts, and these, in turn, opinions, beliefs and true knowledge.

It has already been said that all labors primarily divided themselves into receptive and projective. I now wish to extend this defining a little further, as expressive of this primary division, and say they also divide into negative and positive, or passive and active, cumulative and distributive.

Secondarily, they divide into fractional and whole or unitary, and these into binary and ternary—unitary corresponding to and belonging in the mineral kingdom; binary to the vegetable, and ternary to the animal kingdom.

Thirdly, multitudinous occurring in and properly belonging to man and the superior intelligences.

Each form of labor being an advance upon the preceding in regular numerical order, with an addition of something more elevated and complicated, so that the higher we go, the more ballast we need. Who has not often with interest contemplated the pleasure with which infants and young creatures, when not confined, exert every muscle and organ in their beautiful bodies? They must do the work of projection as fast as the receptive influx has charged them, or they fail of the development intended.

Just how far these works are the subject of consciousness, will depend upon the circumstances attendant upon begetting, gestation, birth and after care; constituting the primals of germinal developmental and digestive education. A failure ever so minute here is sure to produce its infallible impress in the exact ratio of the importance of the arrest of full receptive or projective labor. This law accounts for the scarcity of the typal perfection among animals and man. Also in the trades and professions.

| *Compensation* | |
| --- | --- |
| 1. High.<br>2. Intermediate<br>3. Low. | The highest return is in consciousness of use. Intermediate pay is in the recognition of our abilities, and gratifies ambition. Lowest remuneration is in representative |

of value, cash or its equivalent, and affords us a *solid* basis upon which to build our edifice.

Those who labor for the highest reward are also in the way, in case of failure, of the other grades of compensation. But he who sets his aim at 2d or 3d rate elevation will, on this law, only be entitled to that at which he directed his forces, and those incidentally below.

The great majority of aspirants attain a degree lower than their aim, and hence the rule is to find success of the lowest grades most prevalent among all sorts of laborers, viz: Muscular, mixed and mental; sense of *use*, other than selfish, never obtaining in muscular, seldom in mixed, and only with the minority in mental labors; so we have very few who are really always in the highest exercise of their ability.

Multitudinous labors are made up of a great variety of specific forms that are included in groups, and these divided again into more circumscribed limit, down to the special departments, in which are performed the minutiæ of detail of projective labor where operations are produced.

Each of the departments has its particular atmosphere, which has a marked influence upon the progress of those who fully enter within its limits. Hence the propriety of schools of the departments, where free communion may be enjoyed by all in pursuit of the attainment of these branches. For it is not the measure of the true professional man to have him stand a good examination on all science outside of his speciality; but fail in the fundamental and basal knowledges upon which successful practice is dependent. Hence he who quotes Shakspeare, Byron or Paul de Koch with fluency and accuracy should not take precedence of him who knew nothing of these, but was familiar with the works of Esculapius, Aristotle, Galen, Spallanzini, Harvey, Cuvier, Owen, Ehrenberg, Hassall, Carpenter and Rokistansky (et id omne genus.)

Probably the greatest hindrance to true development is in the excessive desire to obtain and *care* for *property.* Most men restlessly strive for the attainment of their particular *ambition.* And by far the greater part of these workers—laborers—seek property in some form, and usually not for its uses, but simply for its *possession.* Here lies the secret of such universal failure,—the product of the labor of brain or hand is esteemed above the work itself, and hence, long before the goal is attained, we have, in the great majority of cases, fretted to destruction the thing that *gets*, in our exclusive *care* of what is gotten—presenting the ludicrous example of "vaulting ambition o'erleaping itself." Then let us distribute as fast as we attain knowledge or skill, and thus *surely* avoid must, rust or loss by dissipation. Cells flow into and become lost in each other, as to individual identity, to be advanced to higher and finer combinations in elevated uses. So let us be willing to project our ideas in a free interchange to the advancement of the profession, even if we should lose our littleness and identity by the noble exercise.

